# Lung Cancer Screening Access and Cancer Stage at Diagnosis Among American Indian and White Patients

**DOI:** 10.1111/jrh.70207

**Published:** 2026-08-21

**Authors:** Bradford E. Jackson, Madison Uhrin, Stephanie B. Wheeler, Christopher D. Baggett, Lisa P. Spees, Joel A. Begay, Ronny A. Bell, Marc A. Emerson

**Affiliations:** ^1^ Lineberger Comprehensive Cancer Center (LCCC) University of North Carolina at Chapel Hill (UNC‐CH) Chapel Hill North Carolina USA; ^2^ Department of Epidemiology Gillings School of Global Public Health University of North Carolina at Chapel Hill (UNC‐CH) Chapel Hill North Carolina USA; ^3^ Department of Health Policy and Management Gillings School of Global Public Health University of North Carolina At Chapel Hill (UNC‐CH) Chapel Hill North Carolina USA; ^4^ Division of Pharmaceutical Outcomes and Policy Eshelman School of Pharmacy University of North Carolina at Chapel Hill (UNC‐CH) Chapel Hill North Carolina USA

**Keywords:** access to care, American Indian, barriers, barriers to screening, low‐dose CT (LDCT), lung cancer screening

## Abstract

**Purpose:**

Given persistently low rates of lung cancer screening among eligible individuals, we examined racial and geographic differences in proximity to low‐dose computed tomography (LDCT) screening facilities among American Indians and Alaska Natives (AIAN) and White adults in North Carolina.

**Methods:**

We used cancer registry data linked with health insurance claims (2016–2020) to examine a cohort of 18,154 AIAN and NHW lung cancer patients. We calculated straight‐line distances between patient's residential ZIP code and nearest LDCT facility available in the year prior to diagnosis, (within ZIP code, 1–10 miles, or ≥10 miles). Logistic regression models assessed associations between distance and stage at diagnosis (localized vs. non‐localized and distant vs. non‐distant), adjusting for race, age at diagnosis, and health insurance status.

**Findings:**

AIAN were more likely than NHW patients to live ≥10 miles from an LDCT facility (36% vs. 17%) and less often diagnosed at a localized stage (19% vs. 26%). Patients living ≥ 10 miles away had lower odds of localized stage diagnosis (adjusted OR = 0.89; 95% CI: 0.82–0.98) compared to those with access within their residential ZIP code.

**Conclusions:**

Greater distance to LDCT facilities was associated with reduced odds of early‐stage diagnosis. Strategies such as mobile screening units and culturally tailored outreach may improve access and outcomes in underserved AIAN communities.

## 1 Introduction

Cancers of the lung and bronchus have the second highest incidence among male and female American Indian and Alaska Native (AIAN) populations over the past 20 years, second only to prostate and breast cancers. Compared to non‐Hispanic White (NHW) individuals, AIAN populations experience lung cancer incidence nationally, with 2015–2019 age‐adjusted rates of 61.6 per 100,000 compared to 58.2 per 100,000 among NHW individuals in the United States [1]. However, incidence rates vary geographically. In North Carolina, 2014–2018 estimates indicate slightly lower incidence among American Indian populations (60.6 per 100,000) compared to NHW populations (65.8 per 100,000) [2]. Despite this, lung cancer mortality remains higher among AIAN populations nationally (42.3 vs. 37.2 per 100,000) [1], while mortality rates in North Carolina are similar between groups (30.5 vs. 30.1 per 100,000) [3].

Higher lung cancer incidence and mortality among AIAN populations have been linked to a combination of behavioral, environmental, and structural factors. Smoking accounts for approximately 82% of lung cancer cases [4], and AIAN individuals have higher smoking prevalence, with patterns of tobacco use varying across communities due to cultural practices and differences in access to culturally tailored cessation programs [5, 6, 7, 8, 9, 10]. In addition, AIAN communities may face increased exposure to radon, the second leading cause of lung cancer, particularly on tribal lands [8, 11]. Limited awareness of lung cancer screening further contributes to disparities, as individuals who are unaware of screening options may be less likely to undergo early detection. These factors collectively increase the likelihood of diagnosis at later stages, when prognosis is poorer [3, 12].

Early‐stage lung cancer has a substantially more favorable prognosis; however, most lung cancer cases are diagnosed at late‐stage across populations with more frequent late‐stage diagnoses for AIAN [3, 5, 13, 14, 15, 16]. Low‐dose computed tomography (LDCT) screening has been shown to improve early detection and reduce lung cancer mortality by up to 20% [16, 17]. Since 2013, the US Preventive Service Task Force (USPSTF) has recommended annual LDCT screening for high‐risk individuals, defined as adults aged 50–80 years with a ≥ 20 pack‐year smoking history who currently smoke or have quit within the past 15 years. Despite these recommendations, screening uptake remains low, with only an estimated 17% of eligible individuals participating in annual LDCT screening [18]. Screening is higher among individuals with health insurance or pre‐existing chronic lung conditions [17, 19, 20]. Less is known about the geographic availability of LDCT screening facilities and how access may differ across racially and geographically diverse populations. Distance to screening facilities may be particularly relevant in rural areas, where a disproportionate share of screening‐eligible individuals reside, and where public transportation may be limited [21].

To address this gap in knowledge, we conducted a descriptive study to characterize incident lung cancer cases among AIAN and NHW individuals in North Carolina. Specifically, we (1) estimated the distance from individuals’ residential location at diagnosis to the nearest LDCT screening facility available in the year prior to diagnosis, and (2) examined whether greater distance to screening facilities was associated with late‐stage lung cancer diagnoses.

## 2 Methods

### 2.1 Study Population

The present analysis was a retrospective cohort study of recently diagnosed non‐Hispanic AIAN (NHAIAN) and NHW lung cancer patients between 2016 and 2020, in which LDCT facility proximity was assessed prior to the outcome of stage at diagnosis. We used North Carolina cancer registry and Medicare claims data from the University of North Carolina Lineberger Comprehensive Cancer Center’s Cancer Information and Population Health Resource (CIPHR) [22]. In brief, CIPHR combines the clinical and demographic characteristics of patients in the North Carolina Central Cancer Registry (NCCCR) with insurance claims from commercial plans, Medicaid, and Medicare fee‐for‐service and Advantage plans.

Eligible patients were diagnosed between the age of 56 and 81 years with malignant lung cancer (International Classification of Disease Oncology third Edition C34X). We restricted the years of the study period (2016–2020) to coincide with the USPSTF's recommendation for lung cancer screening in December 2013 and Medicare's National Coverage Determination 210.14 which was effective for claims with dates of service on or after February 5, 2015. During the study period, the screening age recommendation was 55–80 years of age. We assessed whether patients were diagnosed between ages 56–81 in February 2016. We selected this age range to ensure that all patients would have been eligible for LDCT screening in the year prior to diagnosis under contemporaneous USPSTF guidelines.

We excluded patients with missing diagnosis dates, who were not identified as NHAIAN or NHW individuals, or whose cancer was not their first or only diagnosis. We also excluded individuals whose cancer was identified from death certificate or autopsy or if they did not have a documented stage at diagnosis. We required that all patients included in the cohort had a North Carolina residential ZIP code at diagnosis. We identified patients based on self‐reported race. Self‐reported racial classifications were obtained from the cancer registry, where registry standards allow for patients to provide up to five race classifications (NAACCR item #160–#164). In line with our previous research [2, 23, 24], we categorized patients as AIAN if any of the five fields for race indicated they were “American Indian, Aleutian, or Eskimo (includes all indigenous populations of the Western hemisphere).” Individuals were classified as White if their primary race was recorded as White and no other race field included American Indian or Alaska Native. Race classification was further restricted by non‐Hispanic ethnicity, in alignment with prior literature.

### 2.2 Exposure

Our exposure of interest was distance to nearest LDCT facility; where Healthcare Common Procedure Coding System codes G0297 and S8022 were used to identify LDCT claims, the corresponding facility location, and the dates of claims submitted from this facility. Specifically, we measured straight‐line distance between centroids of a patient's residential ZIP code at diagnosis to the ZIP code of the nearest facility that provided an LDCT in the year prior to a patient's diagnosis. This was used as a proxy for geographic accessibility. The ZIP code assessment period of one year prior to diagnosis was used to align with the guidelines for annual screening. We operationalized this by identifying the minimum distance between (1) a patient's residential ZIP code at diagnosis, and (2) the list of all ZIP codes where LDCT was rendered in the year prior to a given patient's diagnosis. This maintains the temporality of our patient's ZIP code to a potential provider's ZIP code, as we would not want to consider distances to facilities that did not exist during the time in which a patient could have been screened. Once the minimum distances were identified for the analytic cohort, we evaluated the distribution of distances and categorized them into the following mutually exclusive categories: (1) LDCT within ZIP code, (2) LDCT within 1–10 miles, and (3) LDCT greater than or equal to 10 miles.

### 2.3 Outcomes

Stage at diagnosis was our outcome of interest; we operationalized this as two different binary variables for localized vs. non‐localized SEER summary stage and distant versus non‐distant SEER summary stage. These two outcomes were chosen to evaluate the association between distance to available LDCT facilities and early stage diagnosis as well as to de novo metastatic presentation.

### 2.4 Other Variables

Other clinical and demographic variables of interest measured at diagnosis included patient's age, sex, histology, health insurance coverage at diagnosis, and rurality of residence (rural, urban). Lung cancer histology was dichotomized into non‐small cell lung cancer (NSCLC) and small cell lung cancer (SCLC), where the following histology codes were classified as SCLCs (8041–8045). Insurance coverage at diagnosis was obtained from the cancer registry (NAACCR #630) and classified as private, Medicare, Medicaid, other, and none. Patient's rurality of residence was determined using the Rural Urban Commuting Area (RUCA) codes based on their census tract level using the urban focused classification A (RUCA codes 1, 1.1, 2, 2.1, 3, 4.1, 5.1, 7.1, 8.1, and 10.1) [2].

### 2.5 Data Analysis

We calculated race‐specific distributions of characteristics and present continuous variables with their median and interquartile range (IQR), and categorical variables as frequencies and percentages. We generated a histogram for NHAIAN lung cancer patients to illustrate their proximity to LDCT facilities. We created four categories based on two indicators for whether their residing ZIP code at diagnosis had an LDCT facility in it within the past year at the time of diagnosis, and whether any of the adjacent ZIP codes to their ZIP code at diagnosis had an LDCT facility in it during the year prior to diagnosis. These categories were (1) LDCT not in residing nor adjacent ZIP code; (2) LDCT in adjacent ZIP code; (3) LDCT in residing ZIP code; and (4) LDCT in both adjacent and in ZIP code.

For both localized and distant stage at diagnosis outcomes, we first estimated the unadjusted odds ratios (OR) and corresponding 95% confidence limits (CL) for the association between stage at diagnosis and all study variables. We then estimated the adjusted odds ratios (aOR) and corresponding 95% CLs for stage at diagnosis and proximity to nearest LDCT provider, where in our primary analysis “LDCT facility within ZIP code” was the reference category for our exposure. Our models comprised the a priori selected variables to mitigate confounding of the exposure–outcome relationship: race, sex, age at diagnosis, and insurance at diagnosis, as well as histology and rurality. Age at diagnosis was mean centered and scaled to 5‐years. For sensitivity analyses, we ran an additional multivariable model where we treated the distance variable as a continuous measure that was mean centered and scaled to 5‐miles for interpretability. All data management, figures, and analyses were conducted using SAS v9.4 (SAS Institute, Inc, Cary, North Carolina).

## 3 Results

### 3.1 Cohort Characteristics

**FIGURE 1:**
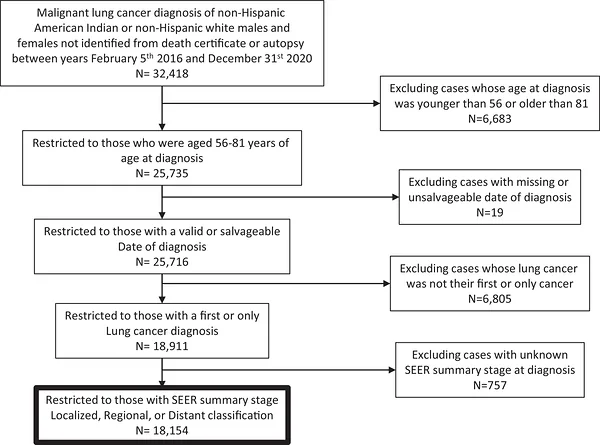
Recently diagnosed lung cancer patient selection diagram.

Our analytic cohort comprised 269 NHAIAN, and 17,885 NHW individuals diagnosed with lung cancer (Figure 1). The frequency of males diagnosed with lung cancer was higher among NHAIAN than NHW (56.13% vs. 51.66%). Table 1 further describes our cohort, where notable distributional differences in health insurance coverage at diagnosis were observed for those with private insurance (NHAIAN: 12.08% vs. NHW: 16.19%) and Medicaid (NHAIAN: 21.13% vs NHW: 12.10%). Fifty five percent of NHAIAN patients resided in rural areas, compared with 28% of NHW. In terms of stage at diagnosis, fewer NHAIAN presented with localized stage at diagnosis (NHAIAN: 18.96% vs. NHW: 25.57%), while both race groups presented frequently and equally with distant stage at diagnosis (NHAIAN: 52.42% vs. NHW: 49.56%).

**TABLE 1:** Demographic and clinical characteristics of non‐Hispanic American Indian and non‐Hispanic White individuals recently diagnosed with Lung Cancer (CCR 2015–2020).

Characteristic		Non‐Hispanic American Indian *N* = 269	%	Non‐Hispanic White *N* = 17,885	%
Sex	Male	151	56.13	9239	51.66
	Female	118	43.87	8646	48.34
Histology	Non‐small cell lung cancer	214	79.55	14830	82.92
	Small cell lung cancer	55	20.45	3055	17.08
Insurance coverage at diagnosis	Private	32	12.08	2863	16.19
	Medicare	154	58.11	11297	63.88
	Medicaid	56	21.13	2140	12.10
	Other	10	3.77	847	4.79
	None	13	4.91	537	3.04
Rurality of residence	Rural	146	55.09	5055	28.34
	Urban	119	44.91	12782	71.66
Age at diagnosis	Median (IQR)	66	62, 72	69	63, 74
	Min, Max	56	81	56	81
SEER stage at diagnosis	Localized	51	18.96	4574	25.57
	Regional	77	28.62	4448	24.87
	Distant	141	52.42	8863	49.56
Distance to nearest LDCT	Median	8.1	0.8, 12.5	5	0, 10.5
	Min, Max	0	67.5	0	75.8
Distance to nearest LDCT	0 miles	84	31.23	5438	30.41
	Between 1 and 9 miles	61	22.68	7613	42.57
	≥ 10 miles	124	46.10	4834	27.03

Abbreviations: IQR, interquartile range; LDCT, low dose computed tomography; SEER, surveillance epidemiology and end results.

### 3.2 Distance to Nearest LDCT Across North Carolina

**FIGURE 2:**
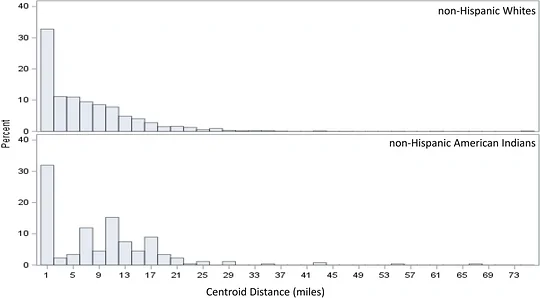
Distribution of centroid distances between ZIP code at diagnosis and nearest LDCT facility in the year prior to diagnosis for non‐Hispanic American Indian and non‐Hispanic White patients.

**FIGURE 3:**
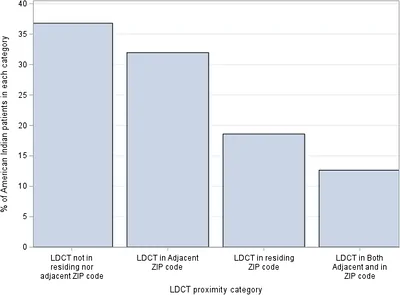
Distribution of the residing and/or adjacent ZIP code having an LDCT facility in the year prior to diagnosis among non‐Hispanic American Indian patients in the analytic cohort (2016–2020).

The bottom of Table 1 and Figure 2 present the race distributions of distance to nearest LDCT on the categorical and continuous scales. Notably, LDCT facilities are more frequently distantly located (i.e., ≥ 10 miles) for NHAIAN patients than NHW patients (NHAIAN: 46.10% vs. NHW: 27.0.3%). Figure 3 presents the distribution of residing and/or adjacent ZIP codes having an LDCT facility in the year prior to diagnosis for NHAIAN lung cancer patients. The majority of NHAIAN patients (68%) did not have an LDCT in their residential ZIP code during the year prior to diagnosis, and only 12% had an LDCT in both their adjacent and residing ZIP code.

### 3.3 Association Between Distance to Nearest LDCT and Stage at Diagnosis

Table 2 presents the ORs for the association with localized stage at diagnosis. When operationalizing distance into three categories, we found that after adjusting for covariates, patients whose ZIP code to ZIP code distance was ≥ 10 miles had 0.89 times the odds (95%CL: 0.82, 0.99) of being diagnosed at localized stage compared with individuals whose residential ZIP code had an LDCT facility located within it in the year prior. The magnitude and direction of the association did not notably change from the unadjusted association to the regression adjusted multivariable models. When operationalizing the distance measure as a continuous variable, mean‐centered and scaled to 5 miles, our model estimated that for every 5‐mile increase above the mean ZIP code to ZIP code distance, there was a decrease in odds of localized stage at diagnosis (aOR = 0.97; 95%CL: 0.94, 0.99).

**TABLE 2:** Odds ratios for associations between patient characteristics, distance to nearest LDCT facility and localized SEER Stage at diagnosis.

		Unadjusted OR (95%CL)	*p* value	Model 1 Adjusted OR (95%CL)^a^	*p* value	Model 2 Adjusted OR (95%CL)^b^	*p* value
Race	NHAIAN vs. NHW	0.68 (0.50, 0.93)	0.0141	0.78 (0.58, 1.07)	0.1233	0.77 (0.57, 1.06)	0.1056
Sex	Female vs. Male	1.28 (1.20, 1.37)	< 0.0001	1.29 (1.21, 1.38)	< 0.0001	1.29 (1.21, 1.38)	< 0.0001
Histology	SCLC vs. NSCLC	0.11 (0.09, 0.13)	< 0.0001	—	—	—	—
Insurance coverage	Private vs. None	1.95 (1.50, 2.54)	< 0.0001	1.98 (1.52, 2.57)	< 0.0001	1.98 (1.52, 2.59)	< 0.0001
	Medicare vs. None	2.59 (2.01, 3.34)		2.22 (1.72, 2.87)		2.22 (1.72, 2.88)	
	Medicaid vs. None	1.67 (1.27, 2.19)		1.64 (1.25, 2.16)		1.64 (1.25, 2.16)	
	Other vs. None	2.52 (1.88, 3.37)		2.51 (1.87, 3.37)		2.51 (1.87, 3.37)	
Rurality	Urban vs. Rural	1.16 (1.07, 1.25)	0.0001	—	—	—	—
Age at diagnosis	Mean centered/scaled to 5 years	1.15 (1.12, 1.18)	< 0.0001	1.10 (1.08, 1.14)	< 0.0001	1.11 (1.08, 1.14)	< 0.0001
Distance to nearest LDCT facility	“1 to < 10 miles” vs. “Within ZIP code”	1.05 (0.97, 1.14)	0.0006	1.04 (0.96, 1.13)	0.0036	—	—
	“≥ 10 miles” vs. “within ZIP code”	0.89 (0.82, 0.98)		0.90 (0.82, 0.99)		—	—
	Mean centered/scaled to 5 miles	—	—	—	—	0.97 (0.94, 0.99)	0.0025

*Note*: — indicates that variable was not included in multivariable model. Model 1, distance to LDCT categorized into proximity categories; Model 2, distance to LDCT treated as continuous measure.

Abbreviations: CL, confidence limit; LDCT, low dose computed tomography; NHAIAN, non‐Hispanic American Indian/Alaska Native; NHW, non‐Hispanic White; NSCLC, non‐small cell lung cancer; OR, odds ratio; SCLC, small cell lung cancer.

Table 3 presents the ORs for the association with distant stage at diagnosis. Neither of the models addressing distance to nearest available LDCT estimated inverse associations. Both models estimated small positive associations, notably for every 5‐mile increase above the mean distance, there was a slight increase in odds of distance stage at diagnosis (aOR = 1.02 95%CL: 1.00, 1.04).

**TABLE 3:** Odds Ratios for associations between patient characteristics, distance to nearest LDCT facility, and distant SEER stage at diagnosis.

		Unadjusted OR (95%CL)	*p* value	Model 1 Adjusted OR (95%CL)	*p* value	Model 2 Adjusted OR (95%CL)	*p* value
Race	NHAIAN vs. NHW	1.12 (0.88, 1.43)	0.3521	1.06 (0.83, 1.35)	0.6511	1.07 (0.83, 1.36)	0.6111
Sex	Female vs. Male	0.80 (0.76, 0.85)	< 0.0001	0.80 (0.76, 0.85)	< 0.0001	0.80 (0.76, 0.85)	< 0.0001
Histology	SCLC vs. NSCLC	3.12 (2.87, 3.40)	< 0.0001	—	—	—	—
Insurance coverage	Private vs. None	0.59 (0.48, 0.71)	< 0.0001	0.58 (0.48, 0.70)	< 0.0001	0.58 (0.48, 0.70)	< 0.0001
	Medicare vs. None	0.47 (0.40, 0.57)		0.53 (0.44, 0.63)		0.53 (0.44, 0.63)	
	Medicaid vs. None	0.63 (0.52, 0.77)		0.64 (0.53, 0.78)		0.64 (0.53, 0.78)	
	Other vs. None	0.50 (0.40, 0.62)		0.49 (0.40, 0.62)		0.49 (0.40, 0.62)	
Rurality	Urban vs. Rural	0.94 (0.88, 1.01)	0.0202	—	—	—	—
Age at diagnosis	Mean centered/scaled to 5 years	0.91 (0.89, 0.93)	< 0.0001	0.93 (0.91, 0.96)	< 0.0001	0.93 (0.91, 0.96)	< 0.0001
Distance to nearest LDCT facility	“1 to < 10 miles” vs. “Within ZIP code”	0.96 (0.90, 1.03)	0.0419	0.96 (0.90, 1.03)	0.0757	—	—
	“≥ 10 miles” vs. “within ZIP code”	1.05 (0.97, 1.14)		1.05 (0.97, 1.13)		—	—
	Mean centered/scaled to 5 miles			—	—	1.02 (1.00, 1.04)	0.0355

*Note*: — indicates that variable was not included in multivariable model. Model 1, distance to LDCT categorized into proximity categories; Model 2, distance to LDCT treated as continuous measure.

Abbreviations: CL, confidence limit; LDCT, low dose computed tomography; NHAIAN, non‐Hispanic American Indian/Alaska Native; NHW, non‐Hispanic White; NSCLC, non‐small cell lung cancer; OR, odds ratio; SCLC, small cell lung cancer.

## 4 Discussion

Disparities in lung cancer outcomes among AIAN populations may be influenced by differences in access to early detection services. This study was undertaken to assess whether geographic access to LDCT screening facilities may be associated with stage at diagnosis. Our findings suggest that geographic proximity to LDCT screening facilities may play a role in early‐stage lung cancer diagnosis, particularly among populations with reduced access to care. Although both NHAIAN and NHW were most frequently diagnosed at later stages, NHAIAN individuals were more likely to reside farther away from LDCT facilities at the time of diagnosis. The observed association between closer proximity and increased likelihood of early‐stage diagnosis, while modest, supports the hypothesis that geographic access to screening may influence early detection. These findings highlight the potential role of structural barriers, including geographic access to care, in contributing to disparities in lung cancer outcomes.

Geographic proximity to screening facilities may influence lung cancer stage at diagnosis through several pathways. Individuals living closer to LDCT facilities may be more likely to engage in screening due to reduced travel burden, increased opportunities for provider referral, and greater integration into healthcare systems offering preventive services. On the other hand, individuals residing farther from screening facilities, particularly in rural areas, may face logistical challenges such as transportation barriers, longer travel times, and limited availability of specialty care [25]. All of which may reduce the likelihood of screening and consequently delay diagnosis, especially given the lack of public transportation in these communities.

Our findings are consistent with a growing body of literature documenting disparities in lung cancer screening access and uptake among AIAN populations. Recent evidence indicates that AIAN individuals have substantially lower LDCT screening completion rates compared to NHW populations, even among those eligible for screening [26]. These disparities have been attributed to a combination of structural barriers, including limited access to screening facilities, gaps in the health care infrastructure, and reduced availability of culturally appropriate care [27]. Regional initiatives, such as the Southeastern American Indian Cancer Health Equity Partnership (SAICEP), have similarly highlighted persistent inequities in access to high‐quality cancer care and elevated cancer‐related mortality among AIAN communities [10]. Together, this body of work suggests that disparities in screening access may contribute to downstream differences in stage at diagnosis and outcomes.

Our findings also align with prior studies examining lung cancer screening behaviors among AIAN populations [12]. Qualitative work by Welch et al. found that limited awareness of LDCT screening and infrequent discussions with healthcare providers contributed to low screening uptake. Similarly, Robichaux et al. reported low rates of shared‐decision making (∼8.5%) and screening completion (∼4%) within an urban Native American health clinic, despite targeted intervention efforts [28]. Notably, screening LDCTs were not available on‐site in that study, requiring external facilities. This underscores how structural barriers, including geographic access to screening facilities, may limit screening uptake even among individuals engaged in healthcare systems.

The geographic patterns observed in our study further underscore disparities in access to LDCT screening in rural regions. Prior research has demonstrated that individuals residing in rural areas are significantly less likely to have access to accredited LDCT screening facilities within reasonable travel distances, and these gaps have persisted over time [25]. This is particularly relevant given that lung cancer incidence and mortality are higher in rural populations with disparities widening as healthcare resources become increasingly concentrated in urban areas [29]. In North Carolina, these patterns are especially prominent in regions such as Robeson County, the largest county in the state where approximately 40% of the county's population is AIAN, and where our analysis suggests relatively limited availability of LDCT facilities. National data further indicate that populations with reduced access to screening are more likely to be diagnosed at later stages, reinforcing the importance of geographic accessibility in early detection [30].

Our findings should be interpreted in light of several limitations. First, we did not have information on individual smoking history, which limited our ability to identify patients who met USPSTF eligibility criteria for LDCT screening. Although smoking is the primary risk factor for lung cancer, our study was focused on access to screening rather than disease etiology; however, prior evidence suggest that smoking behaviors and prevalence is higher among AIAN populations, indicating that a substantial proportion of this cohort may have been eligible for screening [4, 31, 32, 33]. Second, misclassification of race/ethnicity for American Indian is a known limitation in cancer registry data and may have resulted in under‐identification of AIAN individuals [34]. While we used multiple race fields to improve classification, some misclassification may remain. Finally, our measure of distance was based on ZIP code centroids and may not fully capture true travel burden or access to transportation.

These findings suggest that improving geographic access to LDCT may be an important strategy for reducing disparities in lung cancer outcomes. Interventions such as mobile lung cancer screening units, expansion of screening services in underserved areas, and partnerships with community‐based healthcare providers may help address structural barriers to care. Given the disproportionate rural residence of many AIAN populations in North Carolina, targeted and culturally tailored approaches to improve both access to and awareness of lung cancer screening are warranted. More broadly, our results contribute to a growing body of evidence indicating that disparities in lung cancer outcomes are shaped not only by individual‐level risk factors, but also by structural inequities in access to preventive healthcare services.

## Funding

Research reported in this publication was supported by The V Foundation for Cancer Research. CIPHR is supported in part by a National Cancer Institute Cancer Center Core Support Grant P30 CA016086 to the UNC Lineberger Comprehensive Cancer Center. Work was also supported by the Cancer Information and Population Health Resource at the UNC Lineberger Comprehensive Cancer Center, with funding provided by the University Cancer Research Fund via the state of North Carolina.

## Conflicts of Interest

The authors declare no conflicts of interest.

## Data Availability

The authors have nothing to report.
